# Visualization of Moisture Distribution in Stacked Tea Leaves on Process Flow Line Using Hyperspectral Imaging

**DOI:** 10.3390/foods14091551

**Published:** 2025-04-28

**Authors:** Yuying Zhang, Binhui Liao, Mostafa Gouda, Xuelun Luo, Xinbei Song, Yihang Guo, Yingjie Qi, Hui Zeng, Chuangchuang Zhou, Yujie Wang, Jingfei Zhang, Xiaoli Li

**Affiliations:** 1 College of Biosystems Engineering and Food Science, Zhejiang University, Hangzhou 310058, Chinamostafa-gouda@zju.edu.cn (M.G.);; 2 Liandu Agriculture and Rural Bureau, Lishui 323000, China; 3 Department of Nutrition & Food Science, National Research Centre, Dokki, Giza 12622, Egypt; 4 Faculty of Mechanical Engineering, Qilu University of Technology, Jinan 250306, China; 5 Hangzhou Xihu Longjing Tea Organization, Hangzhou 310008, China; xihulongjingcha@163.com (Y.Q.);; 6 Hangzhou Jingle Tea Foundation, Hangzhou 310000, China

**Keywords:** hyperspectral imaging technology, green tea, moisture content, processing procedures, visualization

## Abstract

The distribution of moisture content in stacked tea leaves during processing significantly influences tea quality. Visualizing this moisture distribution is crucial for optimizing processing parameters. In this study, we utilized hyperspectral imaging (HSI) technology combined with machine learning algorithms to evaluate the moisture content and its distribution in the stacked tea leaves in West Lake Longjing and Tencha green tea products during the processing flow line. A spectral quantitative determination model was developed, achieving high accuracy ($R_{p}^{2}$ > 0.940) The model demonstrated strong generalization ability, allowing it to predict moisture content in both types of tea. Through hyperspectral imaging, we visualized moisture distribution in seven key processing steps and observed that moisture content was non-uniform, with the leaf tips and petioles having higher moisture levels than the leaf surface. This study offers a novel solution for real-time moisture monitoring of stacked tea leaves in tea production, ensuring consistent product quality. Future research could focus on refining model transfer techniques and exploring additional tea varieties to further enhance the generalization of the approach.

## 1. Introduction

Tea is one of the world’s most popular beverages [1] and contains a variety of antioxidants that may prevent cancer and diabetes [2,3]. During tea production and processing, the moisture content is an important index for setting processing parameters [4], which affects the physical state and chemical reaction process of tea in-process, and consequently affects the quality of the finished tea [5,6]). The excessive moisture content of tea leaves will lead to a decrease in the content of some key components during subsequent processing, while too low a moisture content will lead to the crushing of tea leaves during processing [7,8]. The visualization of tea moisture content is crucial in the production process. During the spreading phase, visualizing the distribution of moisture content is essential for optimizing processing parameters such as spreading thickness, ambient temperature, and spreading time. Additionally, moisture content distribution plays a significant role in subsequent processes such as fixation, steaming, final panning, and drying. Uneven moisture content distribution, particularly at the edges of the leaves or in the main vein area during processing, can negatively impact the final quality of the tea leaves, resulting in inconsistencies. Therefore, the intuitive visualization of moisture content and its distribution in stacked tea during processing is of great importance for optimizing processing parameters and enhancing the quality of the finished tea.

Traditional tea moisture content testing methods such as gravimetric ovens and moisture analyzers, are more accurate but slower and cannot meet the requirement of real-time online processing [9,10]). Currently, vibrational spectroscopy including visible-near infrared (Vis-NIR), mid-infrared (MIR), Raman, terahertz (THz), and HSI have been widely investigated for the evaluation of tea quality [11]. MIR spectroscopy has better specificity and reproducibility, but it requires more sample preparation and is more affected by moisture [12]. Raman spectroscopy relies on inelastic scattering from the interaction of incident radiations with vibrating molecules. However, the major problem is its low sensitivity due to weak Raman scattering. Therefore, the Raman scattering needs to be enhanced for research [13]. THz waves coincide with the vibration of the hydrogen bond and are vested with a high sensitivity to water molecules. However, this is also the limitation of applying THz spectroscopy to evaluate other compounds in tea leaves with high moisture content [14].

Vis-NIR spectroscopy can reflect the characteristics of molecular multiplicative and combinatorial absorption [15,16,17]. Water is a typical hydrogen-containing group substance, so the Vis-NIR spectroscopy technique is suitable for the moisture content detection of agricultural products [18]. Huang et al. [19] collected Vis-NIR spectroscopy of different batches and varieties of teas and constructed a model for predicting the moisture content of tea using direct standardization combined with partial least-squares regression (PLSR). The $R_{p}^{2}$ of the model was significantly improved from 0.2066 to 0.7595, and the method eliminated the negative effects of sample differences among batches and varieties. HSI combines Vis-NIR spectroscopy and imaging techniques into one system to obtain spectral and spatial information on samples, which has the advantages of being green, fast, and non-destructive [20,21]. In addition, HSI combined with chemometric methods can predict chemical and physical information of samples and can be used for chemical imaging on a single pixel to visualize the distribution of chemical components and to compensate for the limitations of spectroscopic techniques [22,23,24]. Luo et al. [25] established PLSR, least squares support vector regression (LS-SVR), and random forest (RF) prediction models for tea polyphenols and crude fiber using hyperspectral imagery combined with chemometrics. Among them, the PLSR was the best predictor, with predictive coefficients of determination of 0.8 and 0.77, respectively. Wei et al. [26] simulated the actual production environment by using HSI technology, captured the hyperspectral images of the front and back sides of tea leaves on a conveyor belt, and established PLSR and LS-SVR models to predict the moisture content of tea. Tea leaves front and back sides were classified, and a dual-purpose prediction model of moisture content was established.

Although spectroscopic and spectral imaging techniques have been applied in the detection of moisture content in tea, the current research cannot achieve real-time online detection of moisture and its distribution in the stacked tea in-process on the production line. HSI technology combined with machine learning algorithms was adapted to establish a practical and powerful approach for visualization of moisture distribution of stacked tea in-process on the manufacture flow line; furthermore, three varieties of tea trees were selected as research objects for investigation of the dynamic changes in moisture content during processing under two types of tea processing techniques.

## 2. Materials and Methods

### 2.1. Samples Collections and Preparations

Samples during the processing of West Lake Longjing tea were collected from 26 March 2023 to 13 April 2023 at Hangzhou Xihu Longjing Tea Company. The processing of West Lake Longjing includes five procedures, including spreading, first fixation, rehydration, second fixation, and final panning. The spreading process is the initial stage in West Lake Longjing production, aimed at allowing the fresh leaves to lose some moisture, reduce bitterness, and enhance the aroma of the tea. The fixation process is the most critical procedure. West Lake Longjing employs the stir-frying method, utilizing high temperatures to evaporate a certain amount of moisture from the leaves, which serves as the preliminary shaping process. After fixation, the leaves are placed in a cool, shaded area for rehydration, which helps to evenly distribute the moisture within the leaves. The final stage, final panning, involves further stir-frying to dry and shape the leaves, giving them a smooth, flat appearance. According to the actual processing situation in the factory, the same batch of fresh leaves and semi-finished products in the five procedures were sampled, respectively, and a total of 950 samples were collected, including 2 varieties of ‘Longjing 43’ and ‘Quntizhong’, among which there were 235 samples of Longjing 43 and a total of 715 samples of ‘Quntizhong’, and the specific information of the samples is shown in Table 1.

Samples of Tencha tea were collected from 28 April 2023 to 22 May 2023 at Hangzhou Jingle Tea Company. In detail, the production of Tencha tea contains four processing procedures: steaming, drying, stem-leaf separation, and color sorting. Since the stem-leaf separation and color sorting processes have less influence on the moisture content of tea leaves, only the same batch of fresh tea, steaming, and drying procedures were sampled. The Tencha is fixed by steam, where high-temperature steam is applied for a short duration to deactivate the enzymes in the leaves, effectively preserving the chlorophyll. Drying is the final stage of the Tencha, where high temperatures rapidly evaporate moisture from the leaves. This step plays a crucial role in maintaining the stability of the tea’s quality during subsequent storage and transportation. A total of 1126 samples were collected, all of which are of the ‘Jiukeng’ variety, and the specific information about the samples is shown in Table 1.

### 2.2. Hyperspectral Image Acquisition

A hyperspectral camera Specim FX10 (Specim, Spectral Imaging Ltd., Oulu, Finland) with a wavelength range of 400 nm to 1000 nm was used for image acquisition of tea samples. The spatial resolution is 2.8 nm, the number of spectral bands is 951, the effective pixel size is 6.45 × 6.45 μm, the effective slit width is 30 μm, the effective slit length is 14.2 mm, and the relative aperture is F/2.4. The hyperspectral camera was installed on a gantry and spanned the conveyor belt on the processing flow line, and the hyperspectral image information of the tea sample was scanned in line scanning mode along with it moving on the conveyor belt. Before hyperspectral image acquisition, the key parameters of the system were adjusted, including camera focus, lens height, conveyor belt moving speed, camera exposure time, and frame rate, to ensure the acquisition of clear hyperspectral images. The distance between the lens of the camera and the sample was set to 60 cm, the conveyor belt moving speed was set to 7 mm/s, the exposure time was set to 45 s, and the frame rate was set to 20 fps. To eliminate the effects of uneven light distribution and dark current on the hyperspectral images, the dark current information was collected before the acquisition of hyperspectral images of the samples, and a standard strip whiteboard (made of polytetrafluoroethylene) was acquired with the samples. During the collection process, heated samples were cooled down before data was collected, so the temperature was ambient.

### 2.3. Determination of Moisture Content of Tea

**Figure 1:**
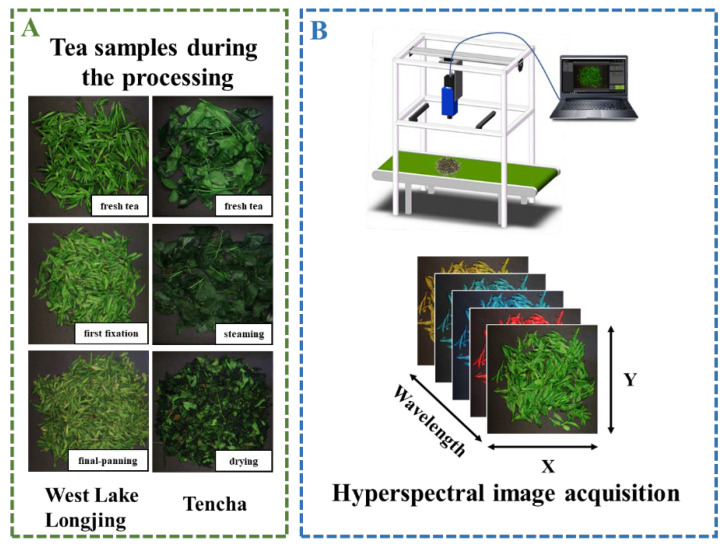
The experimental flowchart of the overall hyperspectral study design. (**A**) Tea samples used for HSI data collection; (**B**) HSI system and obtained data cube.

The standard values of the moisture content of tea were obtained according to Wang et al. [4], and the experimental process is shown in Figure 1. A high-precision electronic balance (Sartorius BSA224S, Gottingen, Germany) with a precision of 0.1 mg was used to determine the weight of the tea leaves, and a hot air oven (STIK BAO-50A, Guangzhou, China) with a control precision of 1 °C was utilized to dry the tea leaves. After that, the moisture contents were recorded according to the following equation:

(1) $$Mositurecontent\left(\%\right)=\frac{\left(W1-W2\right)}{W1}\times 100$$

In Equation (1), W1 is the weight (g) of the sample before drying and W2 is the weight (g) of the sample after drying.

### 2.4. Data Processing

#### 2.4.1. Spectral Pre-Processing

The spectral features of the sample and background were analyzed using ENVI 5.6 (Exelis VIS, Washington, DC, USA), and the corresponding mask image of the sample was constructed in PyCharm using the Python 3.8.13 language. Binarized image segmentation was performed, and the spectra of the sample’s pixel points were finally extracted. The spectra of each pixel point were corrected for black and white according to Equation (1), and the corrected spectra of all pixel points were calculated to obtain the average spectrum as the original spectrum of this sample.

(2) $$R=\frac{R_{raw}-R_{B}}{R_{W}-R_{B}}$$

In Equation (2), R is the corrected spectrum of a single pixel point of the sample, $R_{\mathrm{raw}}$ is the raw spectrum of a single pixel point, $R_{B}$ is the spectrum corresponding to the dark current, and $R_{W}$ is the spectrum of the standard strip whiteboard corresponding to that pixel point.

The average spectra of ‘Longjing 43’, ‘Quntizhong’, and ‘Jiukeng’ in different processing procedures were obtained. Among them, the samples of ‘Longjing 43’ and the ‘Quntizhong’ underwent six processing procedures, including fresh tea, spreading, first fixation, rehydration, second fixation, and final panning. The samples of ‘Jiukeng’ contained three processing procedures, including fresh tea, steaming, and drying.

To effectively eliminate noise from the spectra and retain meaningful information in the raw spectral data, the spectra were preprocessed using multiple scattering correction (MSC), Savitzky–Golay smoothing (SG), and normalization (NOR) and their combinations [27,28,29].

#### 2.4.2. Feature Band Extraction

Since the original hyperspectral data has more dimensions and a large amount of redundant information, which was prone to covariance problems and affected the computational efficiency and accuracy of the model, this study adopts the competitive adaptive reweighted sampling algorithm (CARS) [30] in the original high-dimensional data to select a set of the most representative and informative subset of features to optimize the subsequent modeling and analysis.

CARS is an iterative process to find and select features in the full spectral range, updating the feature weights based on the performance of the partial least squares regression model so that features contributing more to the model performance were given higher weights, while low contributing features were given lower weights. Additionally, based on the ten-fold cross-validation, multiple subsets of variables were obtained through multiple samples, and the subset of variables with the smallest cross-validated root mean square error of validation was selected based on the cross-validation. The selected variables were the set of optimal wavelength variables [31,32,33].

#### 2.4.3. Classification Models and Model Evaluation

The machine learning algorithms of partial least-squares discrimination analysis (PLS-DA) and RF were used to classify different processing procedures for different tea varieties. PLS-DA is a linear classification method that combines the properties of partial least squares regression with the discrimination power of a classification technique [34]. Random forests are a combination of tree predictors such that each tree depends on the values of a random vector sampled independently and with the same distribution for all trees in the forest [35]. RF is robust, handles missing data well, and is less prone to overfitting, making it ideal for tasks such as classifying different tea varieties or processing procedures based on complex datasets. The processing procedures of West Lake Longjing include fresh leaves, spreading, first fixation, rehydration, second fixation, and final panning. The processing procedures of Tencha include fresh leaves, steaming, and drying. The optimal model hyperparameters were obtained using five-fold cross-validation. Accuracy was used as the main evaluation index for the performance of the classification model, and in general, the closer the accuracy is to 1, the better the performance of the model [36]. In addition, a confusion matrix was used to observe the classification of each category of tea.

#### 2.4.4. Regression Model and Model Evaluation

Machine learning algorithms of PLSR and support vector regression (SVR) were used to predict the moisture content of tea. The hyperspectral instrument employed in this study has 224 spectral bands. Therefore, the independent variable dataset *X* consists of rows representing the number of samples and 224 columns corresponding to the spectral bands. The dependent variable dataset *Y* contains a single feature, which represents the actual measured values of tea leaf moisture content, with the number of values equal to the number of samples. In Python, the PLSRegression and SVM classes from the sklearn library were used to construct the PLSR and SVR models, respectively. For both models, the test set size was set to 0.3, and the random state was fixed at 42. The number of principal components in the PLSR model was set to 20. In the SVR model, the kernel parameter was set to ‘rbf,’ *C* to 100, *γ* to 0.1, and *ϵ* to 0.1. The optimal model hyperparameters were obtained using five-fold cross-validation, and the coefficient of determination ($R^{2}$), root mean square error (RMSE), and relative prediction deviation (RPD) were used as the main evaluation index of regression model performance.

$R^{2}$ indicates the goodness of fit of the regression equation generated by the model and was applied to the five-fold cross-validation and test sets, denoted as $R_{\mathrm{cv}}^{2}$ and $R_{p}^{2}$, respectively. RMSE indicates the difference between the predicted and measured values and was applied to the five-fold cross-validation and test sets, denoted as RMSECV and RMSEP, respectively. Usually, the closer $R^{2}$ is to 1, the better the stability of the model; the closer the RMSE is to 0, the higher the accuracy of the model; the larger the RPD is, the better the predictive performance of the model; when the RPD is <1, the model predicts poorly; when 1 ≤ RPD < 2, the model predicts general performance; when 2 ≤ RPD < 3, the model prediction performance is good; when RPD ≥ 3, the model prediction performance is excellent [37].

By plotting scatter plots and histograms, the performance of the regression model can be evaluated more intuitively. The scatterplot was generated by plotting the predicted moisture content values on the *y*-axis and the corresponding measured moisture content values on the *x*-axis. Each point on the scatterplot represents a single sample, with its position determined by the predicted and measured moisture content values. The data were grouped based on the different processing procedures applied to the tea leaves. Each processing procedure (such as spreading, fixation, rehydration, etc.) was treated as a separate group. This grouping allowed us to compare how well the model predicted moisture content at each processing stage, and how the measured values compared to the predicted values for each group.

#### 2.4.5. Generalization Capability of the Model

The regression models were trained separately on each type of data, and their ability to predict moisture content for a different variety was assessed. This process helps to test the adaptability of the model across different tea varieties, simulating a real-world scenario where a model trained on one variety needs to perform well on others. In this study, the samples of one tea type were used as the training set, while the samples of the other type were used as the test set. This was repeated by swapping the training and testing sets to evaluate how well the model generalizes to unseen data from a different variety.

#### 2.4.6. Visualization of Moisture Content

In hyperspectral imaging, each image comprises a spectrum corresponding to every pixel, allowing the creation of a spatial prediction map by applying a developed model to these individual spectra [38]. The optimal tea moisture content prediction model was selected, then the corrected spectra of each pixel point in the sample were imported into the prediction model, and the moisture content of each pixel point was calculated. Subsequently, the spectral data were transformed according to the pseudo-color rules of the predicted values. Distribution maps were generated using pseudo-color scales to visually display the spatial distribution of the stacked tea leaves on processing assembly lines [39,40].

## 3. Results and Discussion

### 3.1. Moisture Content Analysis of Different Processes

**Figure 2:**
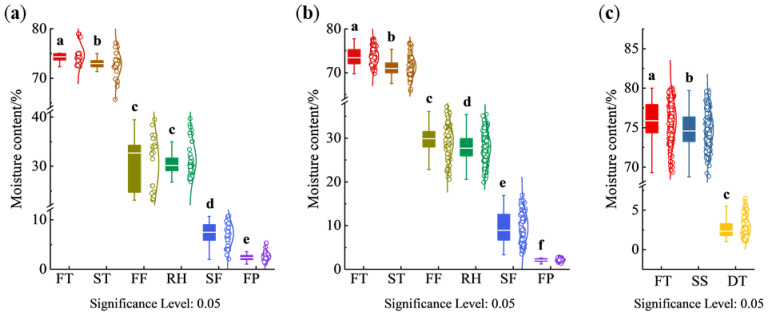
Moisture content variation in different varieties during various processing procedures. (**a**) Longjing 43; (**b**) Quntizhong; (**c**) Jiukeng. FT, fresh tea; ST, spreading; FF, first fixation; RH, rehydration; SF, second fixation; FP, final panning; SS, steaming; DT, drying. Different lowercase letters (a–f) among groups mean statistically significant differences (*p* < 0.05).

The moisture content of tea samples of different varieties and different processing procedures is shown in Figure 2. The tea fresh leaves of West Lake Longjing were made from varieties of Longjing 43 and ‘Quntizhong’, and their processing procedures include spreading, first fixation, rehydration, second fixation, and final panning. The moisture content of tea fresh leaves from both Longjing 43 and ‘Quntizhong’ was concentrated at 72–80%. After spreading, the moisture content decreased, but the decrease was small, which might be affected by the weather. When the air humidity was small, the tea leaves lost moisture faster, so the moisture content of leaves after spreading and after fixation was lower; when the air humidity was large, or when the surface of the leaves showed moisture adsorption due to rainfall, the tea leaves lost moisture slower during the spreading process, so the moisture content of leaves after spreading and after fixation was higher. Comparing Longjing 43 and ‘Quntizhong’, the moisture content of the leaves of Longjing 43 was concentrated at 30–40% after the first fixation, while the moisture content of the leaves of ‘Quntizhong’ was concentrated at 25–35% after the first fixation. Because Longjing 43 germinated earlier, and the exploitation period was about ten days earlier than that of ‘Quntizhong’, and its leaves were slightly larger than those of ‘Quntizhong’, in the process of the first fixation, the temperature of the first fixation of Longjing 43 was lower than that of ‘Quntizhong’ by 2–3 °C and the time of fixation was shortened by 10 s-15 s in order to avoid the rapid moisture loss of leaves and thus the phenomenon of uneven moisture loss, burnt edges, and paste edges. Therefore, the moisture content of Longjing 43 after the first fixation will be higher than that of ‘Quntizhong’. The moisture content of the tea leaves increased after rehydration, and due to the thickness of the stacked tea leaves during the rehydration process, the deeper layer of the leaves was more humid and had a high moisture content, while the surface layer of the leaves was drier and had a low moisture content, so the distribution of the moisture content of the leaves had a larger span. The moisture content of Longjing 43 was distributed at 4–13% after the second fixation, and the moisture content of ‘Quntizhong’ was in the range of 4–18%. After final panning, the moisture content of Longjing 43 and ‘Quntizhong’ was usually in the range of 3–6%, too high moisture content in the semi-made tea will make the chemical components in the tea oxidized; too low moisture content will affect the taste and flavor of the tea.

The fresh leaves of Tencha were made from the ‘Jiukeng’ variety, and its processing procedures included steaming, drying, stem-leaf separation, and color sorting. The moisture content of fresh leaves of ‘Jiukeng’ was distributed at 73–80%. During the steaming procedure, when the leaves were younger, the fixing machine body tilt angle was smaller, and the fixing time was shorter; when the leaves were older, the machine body tilt angle was larger, and the fixing time was longer. Due to the steaming procedure, the surfaces of the leaves were attached to the moisture, so after steaming, the moisture content change of the tea was small, distributed at 70–80%. The moisture content of leaves after drying was in the range of 2–7%.

Through the above analysis, the moisture content of fresh leaves and semi-finished products in the processing procedures fluctuates greatly, so the realization of real-time, accurate, non-destructive detection of moisture content, for the guidance of mechanized processing and standardized production is of great significance [41].

### 3.2. Spectral Analysis

**Figure 3:**
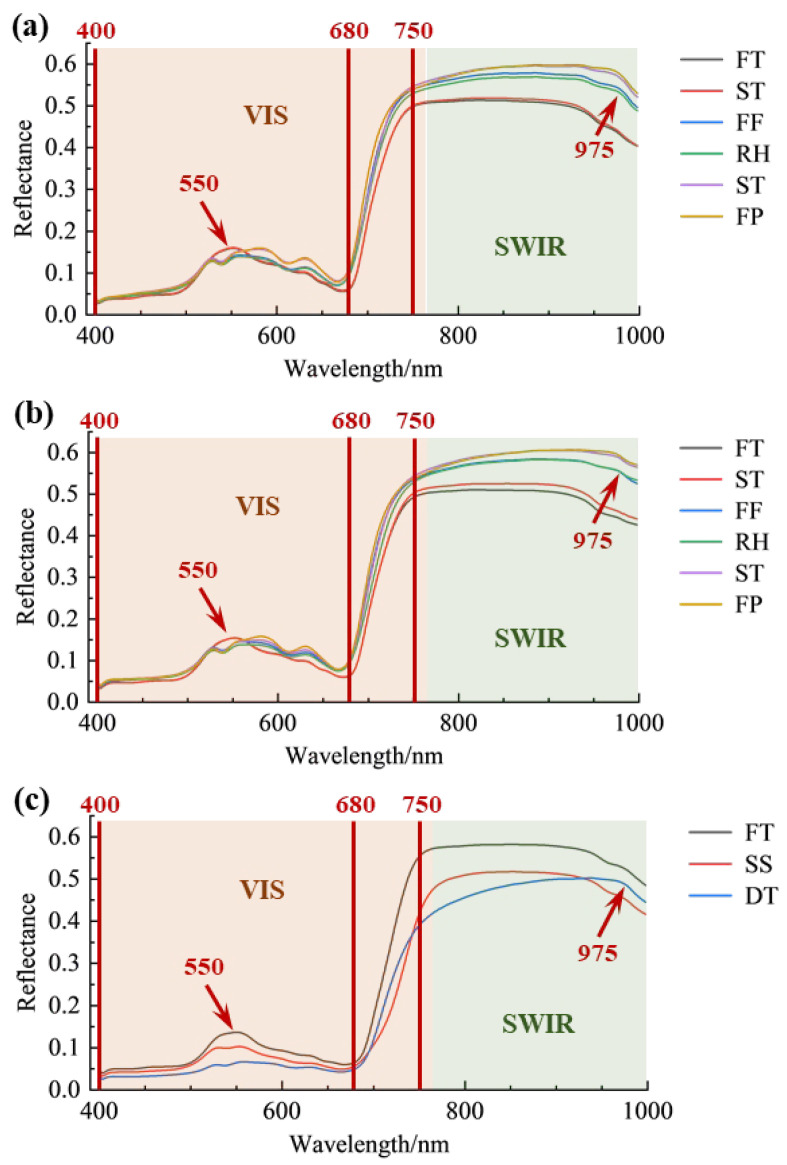
Average spectra of the three varieties during various processing procedures. (**a**) Longjing 43; (**b**) Quntizhong; (**c**) Jiukeng. FT, fresh tea; ST, spreading; FF, first fixation; RH, rehydration; SF, second fixation; FP, final panning; SS, steaming; DT, drying.

The average spectra of the three varieties and various processing procedures are shown in Figure 3. In general, there were several differences in the reflective intensities of the three varieties. Also, the average spectra of the ‘Longjing 43’ and the ‘Quntizhong’ had a very close trend, where the differences in reflectance intensities indicated that the internal composition of the varieties of ‘Quntizhong’ and ‘Longjing 43’ had different contents [42]. Meanwhile, the spectral characteristics of fresh and steamed leaves from ‘Jiukeng’ showed similarities, which can be attributed to the steaming process used for Tencha production. This process, known for its short duration, helps retain chlorophyll and preserve the color of fresh leaves. Additionally, the steaming process results in a slight increase in surface moisture, contributing to a higher overall moisture content in the leaves [43].

In the current study, the low reflectance in the visible spectral range of 400–680 nm was due to the strong absorption of chlorophyll and carotenoids, which were easily excluded from the model calculations based on their instant variations among the studied groups during the drying process compared to the huge variation in the moisture contents that were observed at specific wavelengths [27]. For instance, the varieties of ‘Quntizhong’ and ‘Longjing 43’ had an obvious stable peak at 550 nm which is mainly consistent with the content of chlorophyll. For checking the big change based on the different tea processing procedures, West Lake Longjing was fixed by using a flat tea frying machine that uses a high temperature (200 °C), which makes the fresh leaves change from bright yellowish-green to dark green [44,45], so there was a significant decrease in its sample reflectance at 550 nm after the fixation process compared to the variety of ‘Jiukeng’.

More importantly, the reflectance intensities of leaves in the near-infrared region (780–1000 nm) increased with the decrease in moisture contents, which explained the discontinuities in the internal cellular structure of leaves that might come from the reduction in the enzyme activities of the leaves [46]. The absorbance in the leaves in the near-infrared region (780–1000 nm) decreased with the reduction in moisture content [42]. In addition, an absorption peak appeared at 980 nm, which is assigned to the O-H bond stretching changes of the water molecule that happened during the drying process [47,48,49].

### 3.3. Classification of Different Processing Procedures

It can be found that there are many differences in the spectral reflectance of tea leaves in different processing procedures, so we explore the possibility of spectral reflectance to quantitatively distinguish tea leaves in different processing procedures. Before constructing a quantitative distinguish model, the spectral data were preprocessed differently using MSC, SG, and NOR methods, and PLS-DA and RF models were constructed to classify the processing procedures of tea in-process. According to different types of processing techniques, the samples were divided into two datasets, West Lake Longjing and Tencha, and each processing procedure in each dataset was divided into a training set and a test set in the ratio of 2:1. Fivefold cross-validation was performed in the training set to obtain the optimal model hyperparameters. The quantitatively distinguishing results are shown in Table 2.

**Figure 4:**
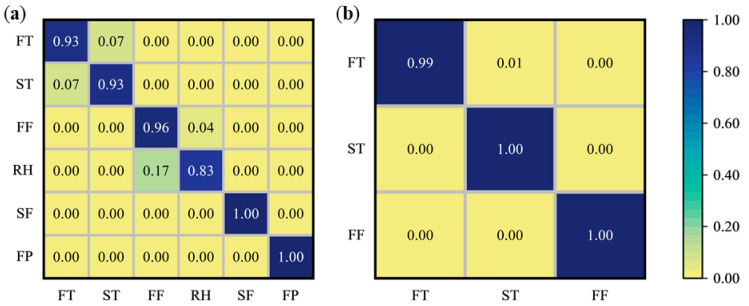
Confusion matrix of the prediction set for the optimal classification model. (**a**) West Lake Longjing; (**b**) Tencha. FT, fresh tea; ST, spreading; FF, first fixation; RH, rehydration; SF, second fixation; FP, final panning; SS, steaming; DT, drying.

In terms of the West Lake Longjing, the classification results of the PLS-DA model with spectra pre-processed by SG had the highest testing accuracy of 94.59. While the RF model with spectra pre-processed by NOR had an accuracy of 90.37%. The prediction accuracy of PLS-DA was higher than that of RF, which may be since the sample sizes of each processing procedure of West Lake Longjing were smaller, and the PLS-DA performed better, while RF needs more data volume to avoid overfitting [50]. For Tencha, the classification results of the model based on NOR combined with RF had the highest testing accuracy of 99.80. While, the testing accuracy of the PLS-DA model after spectral preprocessing by NOR was 99.74%, and the prediction accuracy of the model based on RF was slightly higher than that of PLS-DA. To visually observe the misclassification situation of tea, the best classification models of the two types of tea were outputted in confusion matrix plots, which are shown in Figure 4. In the case of West Lake Longjing, the model can accurately discriminate between the leaves in the second fixation and final panning procedures, while the misclassification occurs between the leaves in the fresh tea and spreading procedures, and the tea leaves in the first fixation and rehydration procedures, which was due to the high spectral overlap of these two groups of procedures. In the case of Tencha, the model can accurately discriminate between leaves after steaming and drying procedures. On the other hand, there was a misjudgment about fresh leaves, which might come from the negative impact of the steaming flow rate on the fixed tea leaves stability. That caused the high similarity between the spectra of the steamed leaves and the fresh leaves.

It is worth noting that spectral technology overcomes the shortcomings of traditional processing techniques that are highly subjective. Spectral data for tea processing provides more objective information about tea semi-finished products, reduces artificial errors in the processing procedures, and can quickly judge the current processing stage based on internal and external attributes of tea in the processing procedures, which contributes to the subsequent quality testing. It can also provide real-time feedback on tea semi-finished products, which helps to adjust the processing procedures promptly and improve the quality of the tea.

### 3.4. Quantitative Determination of Tea Moisture Content

According to the two types of green tea, the samples were divided into two datasets, West Lake Longjing and Tencha. Then, all the sample data are aggregated into one data set. Each dataset was divided into a training set and a test set in the ratio of 2:1. Fivefold cross-validation was performed in the training set to obtain the optimal model hyperparameters. The PLSR and SVR models for the two types and all data were established to predict the moisture content of tea, and the results are shown in Table 3.

It can be found that the $R_{p}^{2}$ values of both PLSR and SVR models were bigger than 0.945 and up to 0.995, the RMSEP was less than 0.640, and the RPD was bigger than 4.115, which indicated that the models had good predictive effects and that it was feasible to establish a prediction model for the moisture content of tea based on hyperspectral technology [37]. There have been many studies on the rapid and non-destructive testing of green tea moisture content. For example, You et al. [51] used computer vision combined with deep learning to detect moisture content during the Tencha drying process, and the correlation coefficient of prediction of the model reached 0.955. Zong et al. [45] used a micro-near infrared spectrometer and portable colorimeter to detect the moisture content of Longjing tea during the full processing process. The correlation coefficient of prediction of the PLSR was 0.982. However, these researches were based on a small sample size, and the generalization ability of these models needs to be improved.

**Figure 5:**
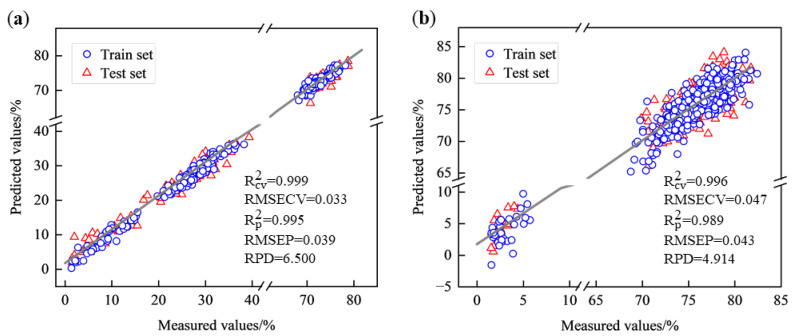
Scatterplot of PLSR model for different types of green tea. (**a**) West Lake Longjing; (**b**) Tencha.

The PLSR models all outperformed the SVR model, which may be due to the relatively linear relationship between spectra and moisture content, which was better captured by PLSR, resulting in better predictions. Therefore, all subsequent analyses were modeled using PLSR. The scatter plots of the PLSR models based on the spectra of West Lake Longjing and Tencha are shown in Figure 5. In the case of West Lake Longjing, the model predictions were more accurate, with $R_{p}^{2}$ reaching 0.999, RMSEP reaching 0.039, and RPD reaching 6.500, and the data in the train set and test set were more uniformly distributed around the 1:1 line.

**Figure 6:**
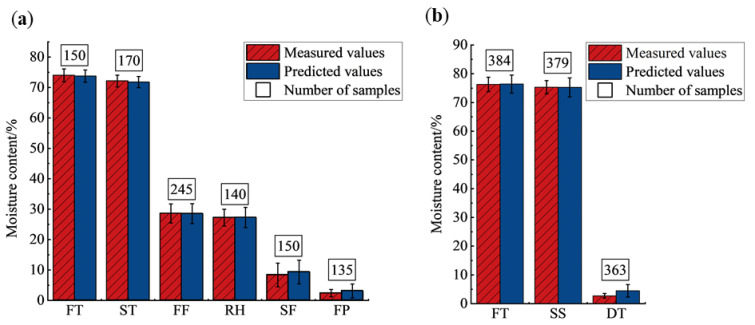
Histograms of measured and predicted moisture content of tea leaves of various processing procedures. (**a**) West Lake Longjing; (**b**) Tencha. FT, fresh tea; ST, spreading; FF, first fixation; RH, rehydration; SF, second fixation; FP, final panning; SS, steaming; DT, drying.

The mean and variance of the measured and predicted values of the moisture content of tea leaves of different types with different processing procedures were calculated as shown in Figure 6. From the figure, there was a decrease in the moisture content of different processing procedures. In the processing of West Lake Longjing, the moisture content of tea decreased significantly after the first fixation and second fixation, and the moisture content of tea decreased slightly during other processing procedures. In the processing of Tencha, the moisture content of tea decreased substantially after drying, and the moisture content decreased slightly after steaming. The predicted and actual values of moisture content of the two different types of tea almost overlapped, indicating that the model has a good prediction effect and can predict the moisture content of different types of tea with different processing procedures.

### 3.5. Generalization Capability of Spectral Determination Model of Moisture

The above analyses relevant models have been established for the datasets of two different types of green tea as well as the mixed dataset. There has been research on rapid and non-destructive testing of tea moisture content during green tea processing. For example, Liu et al. [52] combined machine vision with near-infrared spectroscopy technology to build a quantitative prediction model of the changes in moisture content during the processing of ‘Chuyeqi’ tea, with the correlation coefficient of the prediction set being 0.978. Liu et al. [53] utilized NIR spectroscopy technology for rapid detection of moisture content in the green tea spreading process. The above research focused on a single type of green tea; however, in the actual tea processing, it was necessary to predict different types of tea from the established models. Therefore, the adaptability and transferability of the models should be fully discussed. To investigate the generalization capability of the model among different types of green tea, MSC, SG, and NOR methods and their combinations were used to preprocess the spectra and construct the PLSR model. When the samples of one type were set as the train set for the establishment of the moisture content regression model, while the samples of the other type were set as the test set. The results are shown in Table 4.

It can be found that whether it was West Lake Longjing or Tencha when the model was used for the prediction of the new varieties, the prediction ability of moisture content decreased. When West Lake Longjing was set as the train set and Tencha was set as the test set, the PLSR model with spectra pre-processed by NOR had the best prediction effect, with $R_{p}^{2}$ of 0.778, RMSEP of 0.103, and RPD of 2.121. The PLSR model constructed from the SG preprocessed spectra was the most effective when Tencha was set as the train set and West Lake Longjing was set as the test set, with 0.912 of $R_{p}^{2}$, 0.074 of RMSEP, and 3.378 of RPD. The model performance of the pretreated spectra is better than that of the original spectra, indicating that the performance of the models built by different preprocessed spectra was different, and the spectra can improve the prediction accuracy to a certain extent after preprocessing.

The hyperspectral data used in this study had a total of 951 spectral variables in the range of 400 nm to 1000 nm, which provided rich information but also led to the redundancy of information and decreased computing speed, so CARS was used to select features for the preprocessed full-band spectra, which reduces the interference of other irrelevant components on the spectral signals. The results are shown in Table 5.

**Figure 7:**
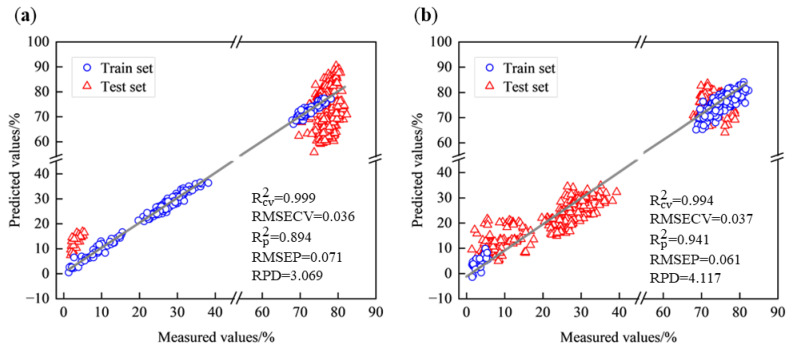
Scatterplot of PLSR model for NOR preprocessing combined with CARS. (**a**) West Lake Longjing modeling predicts Tencha; (**b**) Tencha modeling predicts West Lake Longjing.

After the reduction of dimensionality by CARS, the number of spectral bands was reduced from 951 to 53–103 bands to reduce the redundancy of spectral data. When West Lake Longjing was set as the train set and Tencha was set as the test set, the $R_{p}^{2}$ of the PLSR model constructed after spectra were pre-processed by NOR, and feature selection by CARS was improved from 0.778 to 0.894, the RMSEP was reduced from 0.103 to 0.071, and the RPD was improved from 2.121 to 3.069. When Tencha was set as the train set and West Lake Longjing was set as the test set, the $R_{p}^{2}$ of the PLSR model constructed after spectra were NOR preprocessed and CARS feature selection was improved from 0.599 to 0.941, RMSEP was reduced from 0.159 to 0.061, and RPD was improved from 1.578 to 4.117. This result suggests that the predictive performance of the eigenvariable model was substantially improved compared to the full-band PLSR model and that the PLSR model of NOR preprocessing in combination with CARS exhibits strong generalization when Tencha was set as the train set, which indicates that the spectral characteristics and inclusiveness of the spectral data can vary between different types of tea (Figure 7). The model based on Tencha has better generalization ability and can better predict the moisture content of West Lake Longjing, as ‘Tencha’ may contain specific characteristics that ‘Longjing’ does not have.

### 3.6. Visualization of the Moisture Distribution

**Figure 8:**
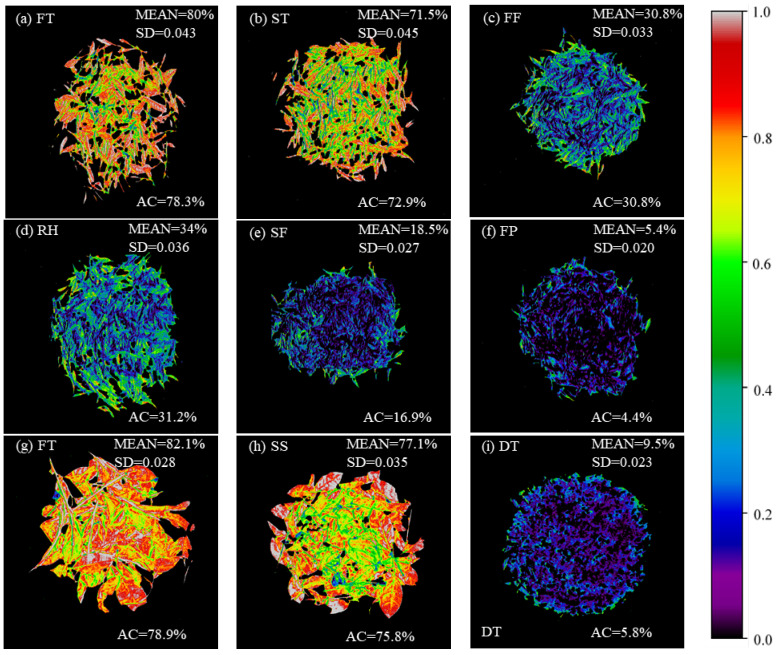
Moisture content distribution of tea in various processing procedures. (**a**–**f**) West Lake Longjing; (**g**–**i**) Tencha. FT, fresh tea; ST, spreading; FF, first fixation; RH, rehydration; SF, second fixation; FP, final panning; SS, steaming; DT, drying. ‘MEAN’ and ‘SD’ represent the mean and standard deviation of the predicted moisture content for all pixel points, respectively, and ‘AC’ represents the measured moisture content of the sample.

The best models for predicting the moisture content of West Lake Longjing and Tencha were selected for visualization of the moisture content of samples of the same batch with different processing procedures, respectively. The visualization of the moisture content distribution of leaves is shown in Figure 8, where different colors represent different contents of moisture, with the black area indicating a lower content of moisture and the red area indicating a higher content of moisture. Along with the processing procedure, the moisture content of the leaves gradually decreased, and the moisture content of the leaves gradually became uniform. Indeed, the visualized distribution of moisture content showed a general decreasing trend that enabled the visual assessment of the time-series changes in moisture content. Also, the visual distribution of moisture content showed a corner effect, where the moisture contents were higher around the perimeter than in the center during the processing procedure [52]. The predicted distribution graph shows that the moisture content of fresh leaves and spreading leaves during the processing of West Lake Longjing was high, in the range of 70–80%. After the first fixation, the moisture content of tea leaves reached 30.8%; and during the rehydration process, the leaves absorbed the water vapor in the air and reached 34%. After the second fixation, the moisture content of the leaves reached 18.5%, and after the final panning, the moisture content of the finished gross tea reached 5.4%. In the processing procedures of Tencha, the moisture content of fresh leaves was 82.1%. After steaming, the moisture content of the leaves reached 77.1%. After drying, the moisture content of leaves reached 9.5%. The prediction results of moisture content were consistent with the measured moisture content, and the standard deviation was less than 0.045, which indicated that the model established in this study was robust and could accurately predict the moisture content of different varieties in their different processing procedures.

Green tea is a non-fermented tea and its processing mainly involves the continuous reduction of water under the action of heat. The moisture content of tea leaves is an important evaluation index in different processing steps. Therefore, the accurate detection of moisture content during green tea processing is very important, which promotes the development of digital and intelligent processing in the tea industry. Zhang et al. [54] established a monitoring model for the contents of green tea processing based on HSI, and the method achieved timely, non-destructive, and accurate detection. Sun et al. [55] combined HSI technology with a combinatorial algorithm to visualize the moisture content in tea leaves. These studies have successfully achieved the prediction and visualization of moisture content in tea leaves based on HSI techniques. In this study, HSI technology and machine learning were combined to demonstrate the distribution of moisture in tea samples during processing. Traditionally, moisture content testing in agricultural processing has relied on wet chemical methods, which are labor-intensive and carry the risk of chemical contamination. Optical detection, such as HSI, as a green and non-polluting analytical technique, provides an optional and ideal solution for online moisture content detection. Therefore, this study provides a sustainable reference model for the detection and visualization of moisture content during tea processing. Meanwhile, the spatial differences in moisture content in different processing procedures and processing uniformity were presented, which helps the processing technicians to adjust the process parameters in time. Furthermore, this approach provides strong support for optimizing the processing procedures, improving the quality of the finished tea, and automating the process. In the current consumer market, consumers are increasingly concerned about the quality of tea, with visualization of the moisture distribution also for producers to gain a competitive advantage in the market, and to promote the tea industry to higher quality and efficiency in the direction of development.

## 4. Conclusions and Future Work

This study demonstrated that combining hyperspectral imaging (HSI) technology with machine learning algorithms effectively classifies tea during processing under various procedures. The PLS-DA model with SG spectra preprocessing achieved a classification accuracy of 94.59% for West Lake Longjing tea, while NOR preprocessing combined with the Random Forest (RF) model achieved 99.80% accuracy for Tencha tea. Additionally, high accuracy in visualizing moisture content distribution was achieved using the CARS feature extraction method and regression analysis. The PLSR model with NOR preprocessing showed strong generalization, with an $R_{p}^{2}$ of 0.941, RMSEP of 0.061, and RPD of 4.117, reflecting robust predictive performance. These results confirm that HSI combined with AI enables real-time monitoring and visualization of moisture distribution, facilitating improved control over processing parameters and enhancing both tea quality and production efficiency.

Future research will focus on investigating other key quality indicators, such as tea polyphenols, amino acids, and caffeine, to further optimize processing parameters. Additionally, exploring long-wave near-infrared spectroscopy to capture more spectral bands related to tea quality is a promising direction. Also, deep learning-based transfer learning strategies deserve to be considered to extend the generalization capability of the constructed models for the generalized detection of different samples. We are also developing an online testing system that will automatically acquire hyperspectral images of semi-finished tea products and provide real-time data on moisture content, distribution, tea variety, harvest period, and origin, to support real-time monitoring and quality control during processing.

## Figures and Tables

**Table 1:** The number of samples from each processing procedure and each variety.

Types	Varieties	Fresh Tea	Spreading	First Fixation/Steaming	Rehydration	Second Fixation	Final Panning/ Drying	Total
West Lake Longjing	Longjing 43	35	50	50	30	40	30	235
	Quntizhong	115	120	165	100	110	105	715
Tencha	Jiukeng	384	0	379	0	0	363	1126

**Table 2:** Model results of spectral classification of various processing procedures.

Varieties	Models	Pretreatment	Accuracy	
			Train/%	Test/%
West Lake Longjing	PLS-DA	None	100.00	92.75
		MSC	100.00	91.47
		SG	99.60	94.59
		NOR	100.00	89.08
	RF	None	100.00	81.93
		MSC	100.00	88.62
		SG	99.60	82.57
		NOR	100.00	90.37
Tencha	PLS-DA	None	99.92	99.54
		MSC	99.94	99.64
		SG	99.87	99.64
		NOR	99.92	99.74
	RF	None	99.95	99.50
		MSC	99.95	99.74
		SG	99.95	99.29
		NOR	99.95	99.80

**Table 3:** Moisture content prediction results for two types of green tea.

**Types**	**Models**	Modeling Results				
		$R_{cv}^{2}$	RMSECV	$R_{p}^{2}$	RMSEP	RPD
West Lake Longjing	PLSR	0.999	0.033	0.995	0.039	6.500
	SVR	0.960	0.051	0.946	0.059	4.323
Tencha	PLSR	0.996	0.047	0.989	0.043	4.914
	SVR	0.956	0.047	0.945	0.049	4.301
All	PLSR	0.996	0.054	0.992	0.054	4.659
	SVR	0.974	0.045	0.972	0.042	5.925

**Table 4:** PLSR full-band model generalization capability based on different preprocessing techniques.

**Train Set/Test Set**	**Pretreatment**	Modeling Results				
		$R_{\mathrm{cv}}^{2}$	RMSECV	$R_{p}^{2}$	RMSEP	RPD
West Lake Longjing /Tencha	None	0.999	0.033	0.455	0.161	1.355
	MSC	0.999	0.037	0.364	0.174	1.254
	SG	0.997	0.033	0.606	0.137	1.592
	NOR	0.998	0.036	0.778	0.103	2.121
	MSC-NOR	0.998	0.036	0.778	0.103	2.121
	SG-NOR	0.997	0.037	0.736	0.112	1.947
Tencha /West Lake Longjing	None	0.996	0.042	0.838	0.101	2.484
	MSC	0.997	0.033	0.500	0.347	0.724
	SG	0.993	0.042	0.912	0.074	3.378
	NOR	0.996	0.037	0.599	0.159	1.578
	MSC-NOR	0.996	0.037	0.599	0.159	1.578
	SG-NOR	0.994	0.037	0.761	0.123	2.046

**Table 5:** PLSR generalization ability based on CARS selection of feature variables and different preprocessing methods.

**Train Set/Test Set**	**Pretreatment**	CVD *	Modeling Results				
			$R_{\mathrm{cv}}^{2}$	RMSECV	$R_{p}^{2}$	RMSEP	RPD
West Lake Longjing/Tencha	None	86	0.999	0.029	0.455	0.161	1.355
	MSC	77	0.999	0.028	0.364	0.174	1.254
	SG	69	0.998	0.029	0.606	0.137	1.592
	NOR	75	0.999	0.036	0.894	0.071	3.069
	MSC-NOR	88	0.999	0.036	0.894	0.071	3.069
	SG-NOR	95	0.998	0.030	0.736	0.112	1.947
Tencha/ West Lake Longjing	None	67	0.994	0.040	0.855	0.096	2.630
	MSC	59	0.995	0.035	0.302	0.189	1.103
	SG	103	0.993	0.040	0.868	0.092	2.748
	NOR	97	0.994	0.037	0.941	0.061	4.117
	MSC-NOR	53	0.994	0.037	0.873	0.090	2.811
	SG-NOR	81	0.993	0.034	0.922	0.070	3.585

* CVD: Characteristic variable dimensions.

## Data Availability

The original contributions presented in the study are included in the article, further inquiries can be directed to the corresponding authors.
